# Juvenile Salmon Usage of the Skeena River Estuary

**DOI:** 10.1371/journal.pone.0118988

**Published:** 2015-03-06

**Authors:** Charmaine Carr-Harris, Allen S. Gottesfeld, Jonathan W. Moore

**Affiliations:** 1 Skeena Fisheries Commission, 3135 Barnes Crescent, Kispiox, British Columbia, Canada; 2 Earth to Ocean Research Group, Simon Fraser University, 8888 University Drive, Burnaby, British Columbia, Canada; University of Toronto, CANADA

## Abstract

Migratory salmon transit estuary habitats on their way out to the ocean but this phase of their life cycle is more poorly understood than other phases. The estuaries of large river systems in particular may support many populations and several species of salmon that originate from throughout the upstream river. The Skeena River of British Columbia, Canada, is a large river system with high salmon population- and species-level diversity. The estuary of the Skeena River is under pressure from industrial development, with two gas liquefaction terminals and a potash loading facility in various stages of environmental review processes, providing motivation for understanding the usage of the estuary by juvenile salmon. We conducted a juvenile salmonid sampling program throughout the Skeena River estuary in 2007 and 2013 to investigate the spatial and temporal distribution of different species and populations of salmon. We captured six species of juvenile anadromous salmonids throughout the estuary in both years, and found that areas proposed for development support some of the highest abundances of some species of salmon. Specifically, the highest abundances of sockeye (both years), Chinook in 2007, and coho salmon in 2013 were captured in areas proposed for development. For example, juvenile sockeye salmon were 2–8 times more abundant in the proposed development areas. Genetic stock assignment demonstrated that the Chinook salmon and most of the sockeye salmon that were captured originated from throughout the Skeena watershed, while some sockeye salmon came from the Nass, Stikine, Southeast Alaska, and coastal systems on the northern and central coasts of British Columbia. These fish support extensive commercial, recreational, and First Nations fisheries throughout the Skeena River and beyond. Our results demonstrate that estuary habitats integrate species and population diversity of salmon, and that if proposed development negatively affects the salmon populations that use the estuary, then numerous fisheries would also be negatively affected.

## Introduction

Estuaries link freshwater and marine habitats for diadromous species such as Pacific salmon (*Oncorhynchus* spp.). Estuaries are staging areas and transition zones where juvenile anadromous salmon can grow rapidly and physiologically adapt to saltwater environments [1–3]. The early marine life history stages, including the period of estuarine residence, are among the most critical life history stages for juvenile salmon [4–8], and growth attained during this period can determine whether they survive to reproduce [9,10]. Despite the emerging appreciation of the importance of the estuary phase to the overall dynamics of salmon populations [5], this phase of the salmon life-history is less well-studied than their marine or freshwater phases [11].

Estuaries provide juvenile salmon with habitats where feeding and growth opportunities are relatively high [1,2] and predation pressure is relatively low. For example, Chinook salmon (*O*. *tshawytscha*) fry grew over 5% a day in the Nanaimo River, BC [1] and restored Puyallup, WA [12] estuaries. Estuary-rearing steelhead (*O*. *mykiss*) grew more rapidly in a seasonally-closed tidal lagoon and exhibited less size-selective mortality than their counterparts that reared in freshwater and went directly to sea [13]. Active feeding and growth in estuaries has been observed even in salmon populations that migrate rapidly seaward [11]. Estuaries can provide cover to juvenile salmonids from predators due to higher turbidity [14], estuarine vegetation, such as seagrass and algae beds [15], and riparian vegetation [16], and rates of predation on juvenile salmon may be lower in estuaries than other habitats. For instance, juvenile Chinook salmon released at estuarine sites were exposed to fewer fish and avian predators than those released to marine sites near Campbell River, BC [17]. Furthermore, while juvenile salmonids were an important food item for common mergansers (*Mergus merganser*) in freshwater habitats they were rarely consumed by mergansers in estuaries [18]. Given the potential importance of estuary food webs and habitats to juvenile salmon, it is perhaps not surprising that juvenile salmon survival rates have been found to be higher in estuaries with less degraded habitat [19].

The duration of estuarine residence varies among anadromous salmon species and populations [11,20,21]. Many populations of coho (*O*. *kisutch*) and sockeye (*O*. *nerka*) salmon may transit rapidly through estuaries [11,20], while others such as chum (*O*. *keta*) and ocean-type Chinook salmon may remain in estuaries for weeks or months [11,20]. Different populations within species also exhibit different timing and patterns of estuarine residence. Juvenile Chinook which enter marine waters in their first year of life may inhabit estuarine habitats for several months [1], while their stream-type counterparts, which rear in freshwater for one year or longer, may occupy estuaries only briefly during their seaward migration [11]. Ocean-type sockeye fry will rear in estuaries where suitable lake habitat is unavailable, such as in the Situk estuary in Alaska where feeding and growth was observed in age-0 sockeye for 3–4 months [20], while age-1 and 2 sockeye swam rapidly through the much larger Bristol Bay estuaries upon ocean entry [14]. Age-0 and age-1 sockeye inhabited brackish waters in the estuary for up to three months in the Chignik AK system where lake-rearing habitat is available [22]. Microchemical analysis combined with daily growth increment counts of otoliths provided evidence of overwintering in estuaries for juvenile coho from two systems in Cook Inlet, AK [23]. Increased estuarine rearing opportunities following estuary restoration in the Salmon River system in Oregon had increased life-history variability among juvenile Chinook salmon that utilized these habitats [21] which supported five different previously described ecotypes [24] ranging from immediate ocean entry to prolonged estuary rearing types. There is continued need to understand how estuary habitats support different species and populations of salmon, particularly for the estuaries of large watersheds with high salmon biodiversity [25].

Hundreds of millions of salmon smolts from a variety of populations and species funnel through the estuaries of large watersheds [11] such as the estuary of the Skeena River, British Columbia, Canada. All species of eastern Pacific salmon and steelhead spawn throughout this 55,000 km^2^ watershed, representing hundreds of distinct populations including up to 70 sockeye, 55 Chinook, 133 coho, 75 even-year pink (*O*. *gorbuscha*), 81 odd-year pink, and 34 chum (*O*. *keta*) salmon populations associated with specific spawning areas [26]. There is considerable genetic, phenotypic and life-history diversity among the different populations of each species, encompassing variation in run timing, age structure, and preferred spawning habitats [27]. Salmon escapements to the Skeena River included approximately 668,000 sockeye, 2.5 million pink, 88,000 coho, and 36,000 Chinook salmon in 2009 [28]. The total returns are higher when the various fisheries are taken into account—the Northern Boundary Technical Committee of the Pacific Salmon Commission estimate an average run size of nearly 3,000,000 sockeye salmon (1985–2012) with an average exploitation rate of 41% [28], and an average exploitation rate for Chinook salmon of about 50% [28]. Chum salmon are the least numerous of the commercially-exploited anadromous species, with estimated escapements of several thousand in recent years [29], considerably less than historical abundances [30]. Steelhead returns to the Skeena River during the past decade have been between 20,000 and 50,000 [31]. These different salmon species support Canadian and USA commercial fisheries, both tidal and freshwater recreational fisheries, and numerous First Nations food, social and ceremonial (FSC) fisheries that occur throughout the watershed. During the peak of the commercial fishing industry in the early 1900s, millions of salmon were captured annually by seine and gillnet fleets that supported dozens of fish canneries in the Skeena estuary [32,33]. Variability of these salmon populations and unpredictable returns now threatens fisheries; for instance low sockeye salmon (*O*. *nerka*) returns in 2013 led to the unprecedented closure of Skeena commercial, recreational, and First Nations fisheries due to conservation concerns [28].

The high salmon biodiversity of the Skeena River system necessarily passes through the downstream estuary during their seaward migration, but it is thought that the duration of estuary residence and resource utilization varies among the different species and populations of salmon [11,20]. For example, pink and chum salmon enter marine waters immediately after emergence at 30–40 mm in length [7] and feed on small zooplankton such as calanoid copepods in nearshore littoral habitats [34]. Coho salmon, which spend one or two years in freshwater prior to their downstream migration, are partially piscivorous when they arrive at sea, sometimes preying on juvenile pink and chum salmon in addition to larval smelt and sand lance in the estuary [35]. In addition to the Skeena River, salmon from other watersheds such as the Nass River and several smaller coastal systems in the region may also utilize the Skeena River estuary. While the freshwater life-history stages of many populations of Skeena River salmon have been extensively studied [27,36–38], there have been comparatively few studies of juvenile salmonids in the Skeena River estuary. The federal Department of Fisheries conducted a survey of juvenile salmon in the Skeena estuary in 1955 [39], and the British Columbia Ministry of Environment conducted a biological assessment of aquatic resources in the Skeena estuary in 1972 [40]. During both of these surveys, thousands of juvenile salmon were captured by beach and purse seine and trawl sampling and juvenile salmon were observed in all parts of the estuary that were sampled. More recently, the Skeena Fisheries Commission conducted a large-scale juvenile salmon sampling project throughout the Skeena estuary as part of a baseline sea lice research project from 2004–2007 [41–43], and also observed juvenile salmon in all parts of the estuary that were surveyed. Thus, while there has been some historic research on juvenile salmon in the Skeena River estuary, it is relatively understudied compared to other large salmon-bearing rivers.

**Fig 1:**
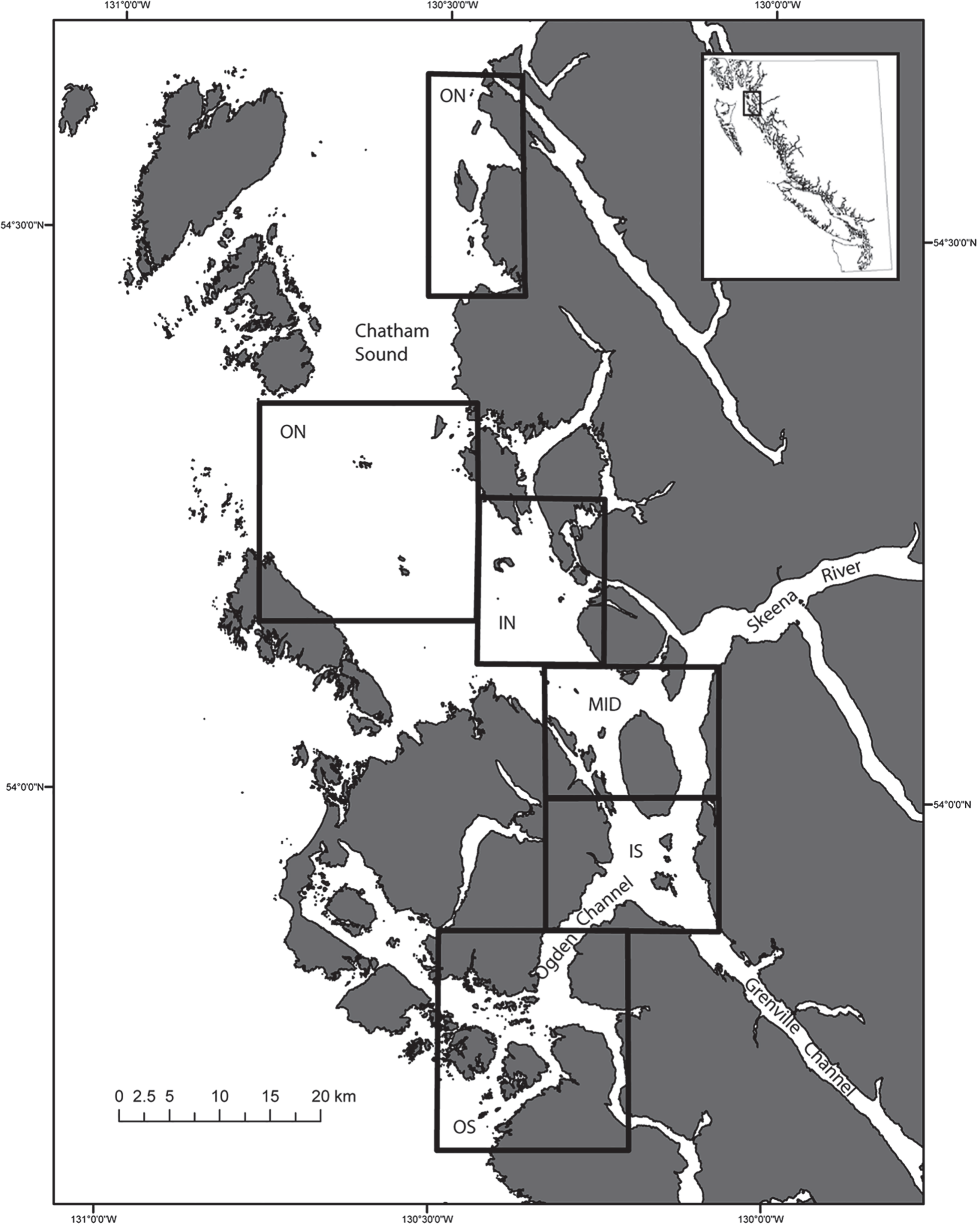
The Skeena River estuary, proposed development, and distribution of juvenile salmon sampling. During the period of highest flow, the zone of freshwater influence extends from the mouth of the Skeena south to Ogden and Grenville Channels, and northwest through Chatham Sound, which also receives freshwater from the Nass River. The study area is shown divided into our analysis regions indicated by the letters IN for inside North, ON for outside north, MID for middle, IS for inside south, and OS for outside south. The IN region contains the focal industrial developments. Note that the ON region includes two polygons.

**Fig 2:**
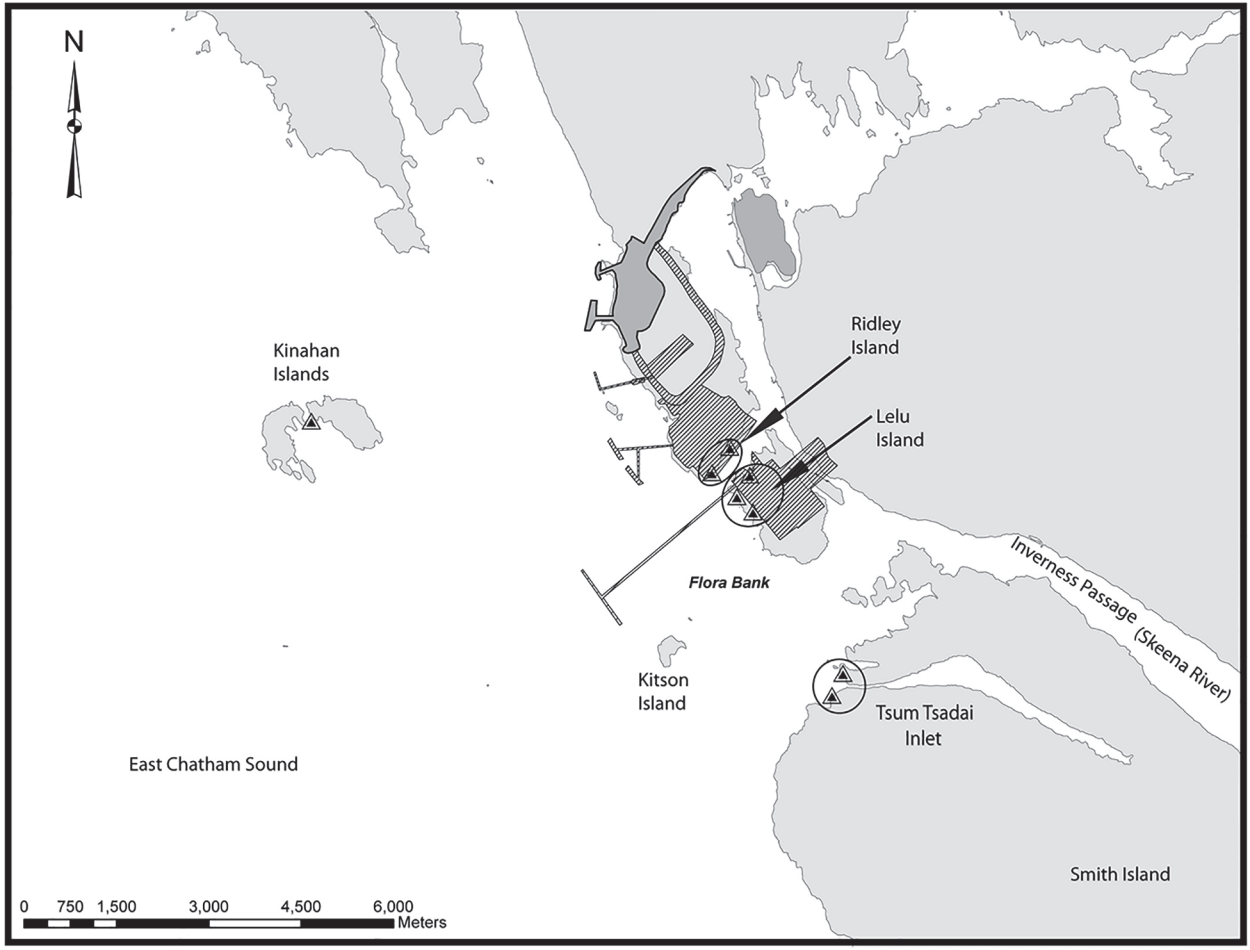
Beach seine sampling stations within the IN region indicated in Fig. 1. Existing developments are shown in dark grey, while proposed development areas are diagonally shaded. Beach seine sampling stations are indicated by triangles. Beach seine sub-regions are indicated with open circles, except at Kinahan Islands where there was only one site.

There are currently several large-scale industrial development projects pending in the Skeena River estuary, including a bulk potash loading facility and two liquefied natural gas (LNG) terminals [44–46] (Figs. 1, 2). The causeway and berth for one of the proposed LNG terminals is situated between Lelu and Kitson Islands on Flora Bank, which represents 50–60% of tidal and subtidal eelgrass habitat in the Skeena estuary. As part of the application process for industrial development, project proponents are required to submit environmental assessments of ecosystem components that could be adversely impacted by the proposed development. Environmental assessment studies conducted by project proponents provide an opportunity to collect information on important ecosystem components such as juvenile salmon and their habitats. However, the scope and time frames of environmental reviews, as well as the number of projects that must complete a federal environmental assessment, have been recently reduced [47]. Previous understanding of estuaries in general [25] and the Skeena River estuary in particular [39,40] suggest that these habitats support juvenile salmon. For example, Flora Banks was previously found to be among the most important early marine habitats for pink salmon from the Skeena watershed, and past proposals for industrial development in the vicinity of Flora Banks were rejected because of concerns about the potential environmental risks to salmon productivity [48]. However, consulting agencies on behalf of the project proponents have submitted environmental assessment applications to the Canadian Environmental Assessment (CEA) Agency for approval of these projects [45,46] without conducting field studies of juvenile salmon. Despite the lack of data from field studies of juvenile salmon, environmental assessment applications have consistently come to the conclusion that proposed projects will have no significant residual negative impacts on salmon populations [49]. At the time of writing, the proposed potash terminal had completed the CEA Agency’s review process under the old legislation and was approved to proceed to the permitting stages and the two LNG terminals entered the environmental assessment process under the new regulations. There is thus a pressing need for scientific data on the usage of the Skeena River estuary by juvenile salmon.

Here we examined the usage of the Skeena River estuary by juvenile salmon. In particular, we examined the geographic and temporal habitat utilization of juvenile anadromous salmon in the greater Skeena estuary in relation to the footprints of the proposed industrial development projects. Furthermore, we used genetic identification to illuminate connections between the estuary habitat and the specific population of origin. These data can help illuminate the current status of salmon biodiversity in the Skeena estuary, and guide decisions regarding its future.

## Methods

### Study area

The main stem of the Skeena River is approximately 570 km long with a mean discharge of about 1,750 m^3^/s. The Skeena River enters the ocean near the village of Port Edward on the north-west coast of British Columbia, where it divides into three channels at a group of islands near the mouth of the river. All of the proposed developments fall within the jurisdiction of the Prince Rupert Port Authority and are located near the exit of the northernmost and central channels, both of which flow northward. At peak discharge, the zone of freshwater influence extends well past this area, approximately 50 km southwest through Ogden Channel, and over 85 km northwest through Chatham Sound and out Dixon Entrance (Figs. 1 and 2).

### Fish sampling

We collected juvenile salmonids by trawl between May 26 and July 5, 2007, and from May 5 to July 1, 2013. Trawl sampling was conducted using a modified trawl which was fished from an 11 m ex-gillnet vessel, HMV Pacific Coast. The trawl net was 18 m long with an opening 5 m wide and 4.6 m deep, with a rigid, baffled holding box designed for live capture [42], and sufficient flotation to maintain a position at top of the water column while fishing. Therefore, only the surface layer of the water column was sampled for all trawls, where juvenile salmon are known to feed during the daylight hours when we sampled [14,50]. The trawl net was deployed for a targeted duration of at least 15 min and up to 20 min for an approximate tow length of 1 km depending on the velocity of prevailing currents. All trawls were conducted within 1 km from shore over water depths ranging from approximately 5 m to over 800 m. Trawl sites were aggregated into generalized regions according to their relative proximity to the northern or southern exit of the Skeena River (Fig. 1). The 2007 trawl sampling program was more extensive than in 2013, and encompassed five regions (Inside North (IN), Outside North (ON), Middle (MID), Inside South (IS), and Outside South (OS)), while the 2013 program encompassed only three regions (IN, IS, and OS) (Fig. 1). Hereafter we refer to these as “regions”. The IN region contains the proposed industrial development footprints.

Beach seine sampling was carried out from April 29 to June 13, 2013 to sample the nearshore fish community. Beach seining occurred weekly at shoreline sites close to proposed industrial activities near the northern entrance to the Skeena River (Fig. 2). The beach seine net was 35 m long and 3 m deep, with 13 mm mesh at the tow ends and 6 mm mesh at the bunt. Each beach seine sampling event consisted of a single set, during which the seine net was deployed down-current from an anchor point on the beach using a 3 m vessel. The beach seine sites were all located within the IN region and were grouped according to the island or inlet where each site was located (Fig. 2). Hereafter we refer to these as “sub-regions”. The Ridley Island and Lelu Island sub-regions are within proposed industrial development footprints.

Average beach seine catches for each species were calculated for each sub-region and sampling week. Trawl catches were normalized based on trawl duration by multiplying the catch by typical duration (20 min) and dividing by the observed duration to obtain a catch per unit effort (CPUE). Average normalized trawl catches were calculated for each species and trawl region.

### Statistical analysis

We analyzed trawl CPUE for sockeye, coho and Chinook salmon with generalized additive models [51]. Specifically, generalized additive models were constructed to estimate the overall mean CPUE for each species in each region in each year by applying a non-parametric smooth function to day-of-year and treating the different regions as parametric factors. The resulting model is of the form

Y=f(d)+β(x)

where Y is the CPUE (mean normalized catch per 20 min set) for a given species, f is a thin-plate regression spline smoother [52] for day of year d, and the β coefficient is the mean abundance for each region x. In essence, these models examine the relative effect of each region on catch rate, after controlling for time. We ran a separate model for each species and each year using a negative binomial distribution with a log link. β is thus an estimate of the relative CPUE of each region on day 0, and is on a log-scale. We used the fitted models to predict the relative abundances of each species at regular intervals in each region during the sampling period, which were back transformed to produce estimates of the CPUE at each region for each prediction interval. To determine the relative support for including region in the model, we used Akaike’s Information Criterion (AIC) to compare the model for each species-year combination with an analogous model that excluded region. Generalized additive models were constructed using the mgcv package [51] in the R programming environment [53].

### Genetic analysis

We used genetic stock identification to determine the spawning location of origin of estuary-caught juvenile salmon. Specific populations of salmon can be separated by their degree of genetic differentiation, which varies according to gene flow; i.e. the rate of migration between populations [54]. Thus the genetic structures of the different pink, chum and coho salmon populations, which have higher straying rates, are less well defined than for Chinook and sockeye salmon populations [55]. At present, there are 29 Chinook and 29 sockeye salmon populations from the Skeena for which baseline genetic data are available that can be reliably separated using microsatellite variation [56,57]. These populations represent geographically and genetically distinct spawning stocks throughout the Skeena watershed, and the baselines are continually modified as new populations are added [58]. The 29 Skeena sockeye salmon populations of the genetic baseline includes populations from 15 different lakes and four river-type populations. Some lakes contain more than one population. For example, Babine Lake, the largest sockeye salmon rearing lake in British Columbia, accounts for up to 90 percent of Skeena River sockeye salmon production and contains at least ten known populations. Populations are spatially related, such that multiple populations from a single lake are more closely related than populations from different lakes. Thus the overall population structure groups the different populations into reporting units that roughly cluster the populations within the different rearing lakes [58].

Tissue samples were collected for DNA analysis from a subsample of Chinook and sockeye salmon specimens. Small pieces of the upper caudal fins were preserved by desiccation on filter paper. DNA was extracted and amplified by polymerase chain reaction [59] at 13 microsatellite loci for Chinook salmon (*Ots2*, *Ots9*, *Ots100*, *Ots101*, *Ots102*, *Ots104*, *Ots107*, *Ssa197*, *Ogo2*, *Ogo4*, *Oke4*, *Oki100*, *Omy325*) [57] and 14 microsatellite loci for sockeye salmon (*Ots2*, *Ots3*, *Ots100*, *Ots103*, *Ots107*, *Ots108*, *Ok1a*, *Oki1b*, *Oki6*, *Oki10*, *Oki16*, *Oki29*, *One8*, *Omy77*) [56]. The polymerase chain reaction products were size-fractionated on denaturing polyacrylamide gels, and allele sizes were determined with an ABI 3730 capillary DNA sequencer. Genotypes were scored with GeneMapper software v3.0 (Applied Biosystems) using an internal lane sizing standard [56]. Allele frequencies were compared with coast wide baselines of 243 sockeye salmon populations from 20 regions, and 207 Chinook salmon populations from 39 regions using a Bayesian procedure [60], in which individual probabilities and stock proportions were assigned using a modified, C-based version of the program BAYES [61]. Genetic analyses were performed at Molecular Genetics Laboratory of Fisheries and Oceans Canada at the Pacific Biological Station.

The population of origin for each specimen was determined based on the geographic distribution of the most likely genetic assignments. We accepted individual assignments above a probability threshold of 90%. Where the probability of assignment to a specific population was less than 90%, we assigned populations to coarser resolution groups of lake, sub-basin, basin, or larger areas depending on the geographic distribution of the five most likely population assignments. Genetic resolution is expected to improve as the baseline is expanded [58].

All fish sampling activities were conducted under a license to fish for scientific, experimental or education purposes issued by Fisheries and Oceans Canada. Fish sampling activities were approved by Simon Fraser University’s Animal Care Committee.

## Results

### Spatial and temporal distribution of juvenile salmonids

We captured juvenile salmonids at all trawl and beach seine sites that were surveyed. Numerous non‐target species were also caught, of which Pacific herring (*Clupea pallasii*) and surf smelt (*Hypomesus pretiosus*) were the most numerous. The total catches by surface trawl were 733 juvenile sockeye salmon, 180 coho salmon, 149 Chinook salmon, 186 pink salmon, 8 chum salmon and 4 steelhead in 2007, and 567 juvenile sockeye salmon, 96 coho salmon, 23 Chinook salmon, 50 pink salmon, and 3 steelhead in 2013. The 2013 total beach seine catch was 132 coho salmon, 11 Chinook salmon, over 250 chum salmon, and thousands of juvenile pink salmon. These data are provided via supplemental materials (S1–S5 Tables).

**Fig 3:**
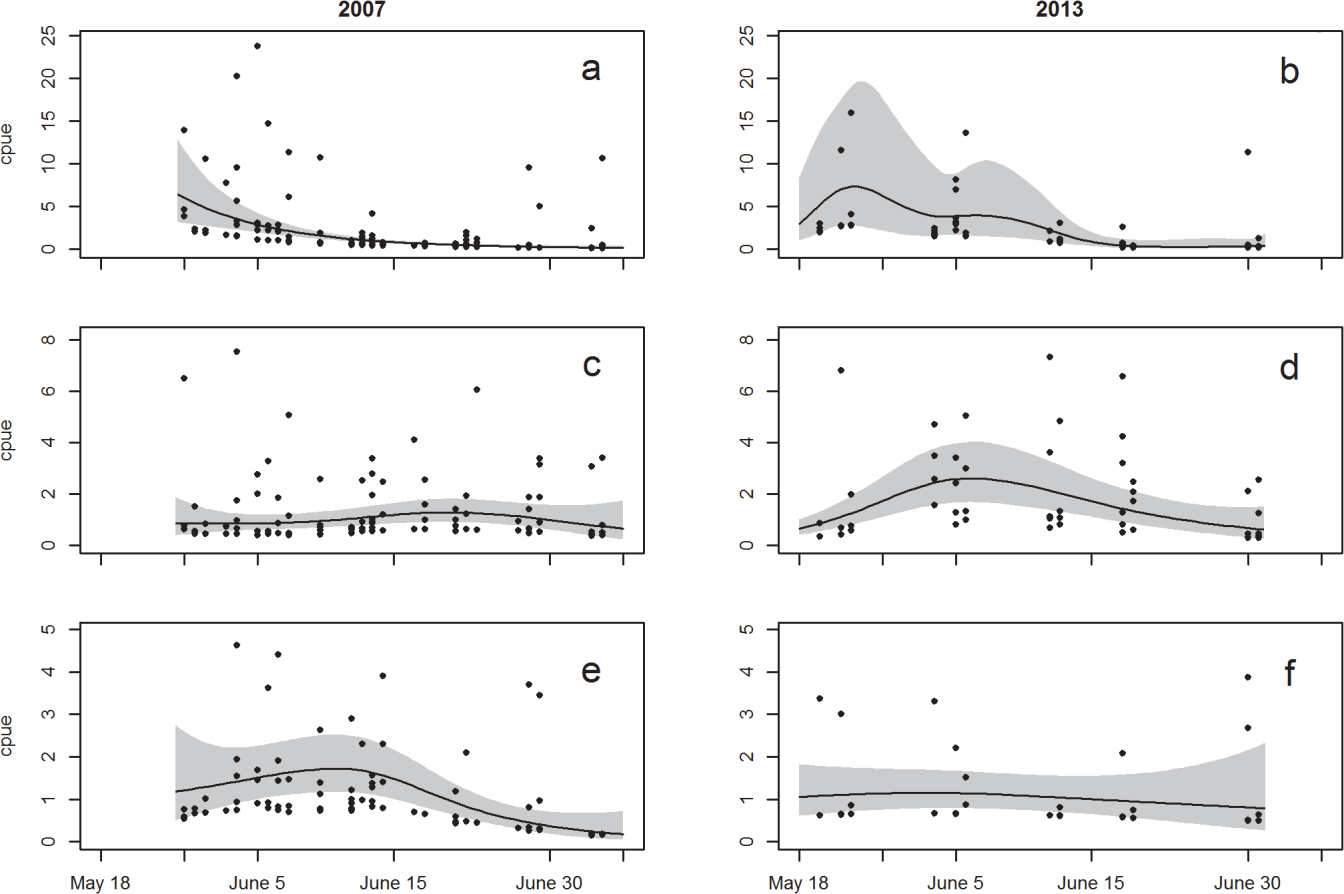
GAM estimates of abundance showing temporal trend for sockeye (a, b), coho (c, d) and Chinook (e, f) salmon abundance during juvenile outmigration season in 2007 and 2013. Points indicate normalized trawl catch per 20 min set, note different scales for each species. The smoothed line and shaded region indicate the temporal trend and confidence region for the GAM models.

Temporal patterns of abundance varied among the different species of salmon captured in the Skeena estuary. High abundances of juvenile pink salmon were observed during early‐season beach seine sets, and were captured in diminishing abundance from the first day of sampling until the second week of May 2013. The highest abundances of juvenile chum salmon were captured by beach seine between the second and fourth weeks of May. Smaller numbers of pink and chum salmon were captured by trawl in 2007, and pink but not chum salmon in 2013. Juvenile coho salmon were observed in trawls from the middle of May onward in both years, and in high abundances in beach seine sets in the third and fourth weeks of May 2013. Juvenile Chinook salmon were captured throughout the sampling period in both years and by both gear types in 2013, and with higher abundances in 2007 than in 2013. Juvenile sockeye salmon, which were only captured by trawl, were the most abundant species captured by trawl in both 2007 and 2013. Sockeye salmon were continually present in the study area from May 26 (the first day of sampling) to July 5 in 2007, and from May 13 until July 1 (the last day of sampling) in 2013, with peak abundances observed between the last week of May and the first week of June in both years (Fig. 3).

**Fig 4:**
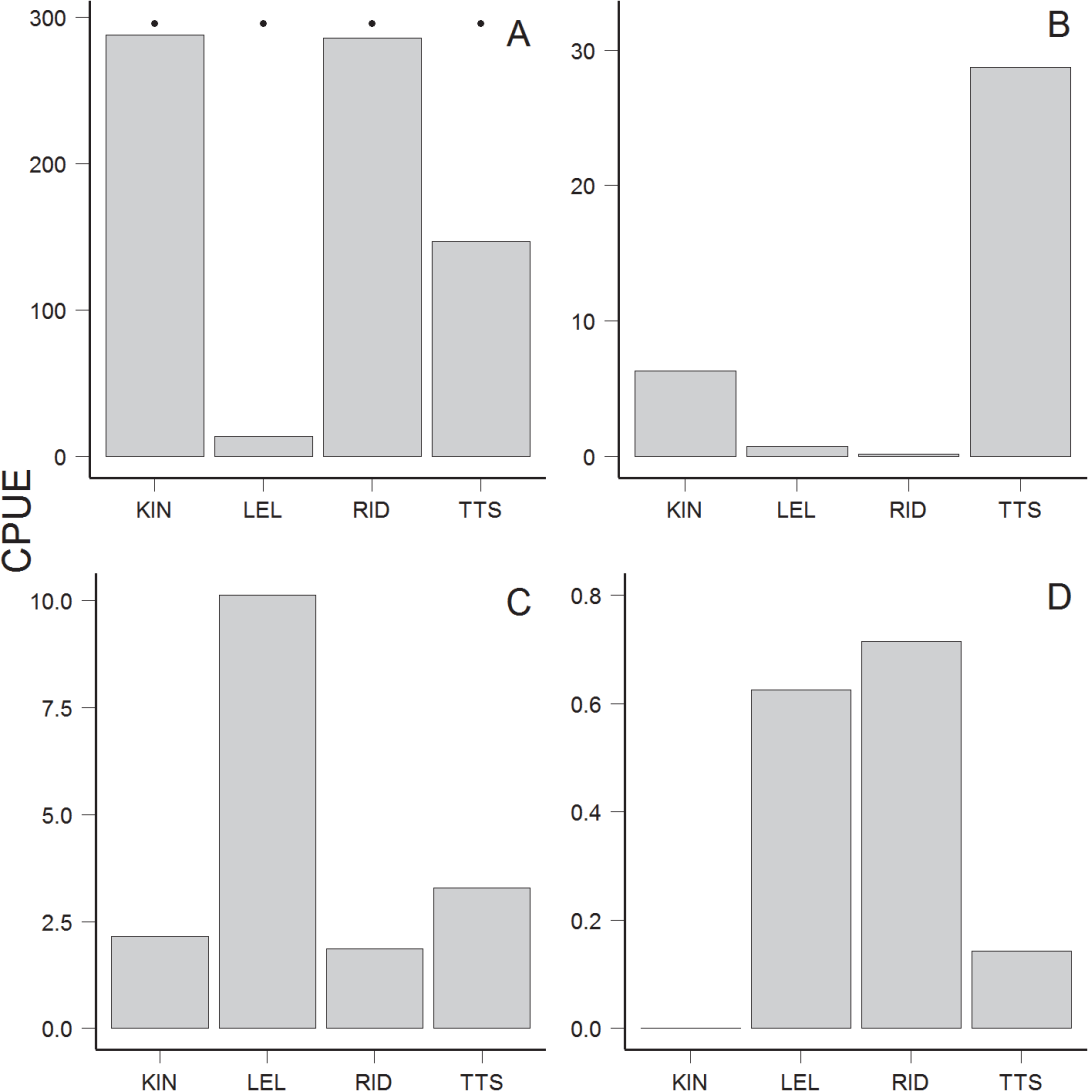
Average beach seine catches of juvenile pink (a), chum (b), coho (c), and Chinook (d) salmon by sub-region, pooled across all sampling dates. No sockeye salmon were captured by beach seine. Pink salmon catches greater than 100 per location are indicated by black dots above bars. Catches greater than 100 or 1000 individuals were calculated as 100 or 1000. Note different scales of y‐axes. Locations are as follows: KIN = Kinahan Islands, LEL = Lelu Island, RID = Ridley Island, TTS = Tsum Tsadai Inlet. LEL and RID sites are within footprints of proposed development.

**Fig 5:**
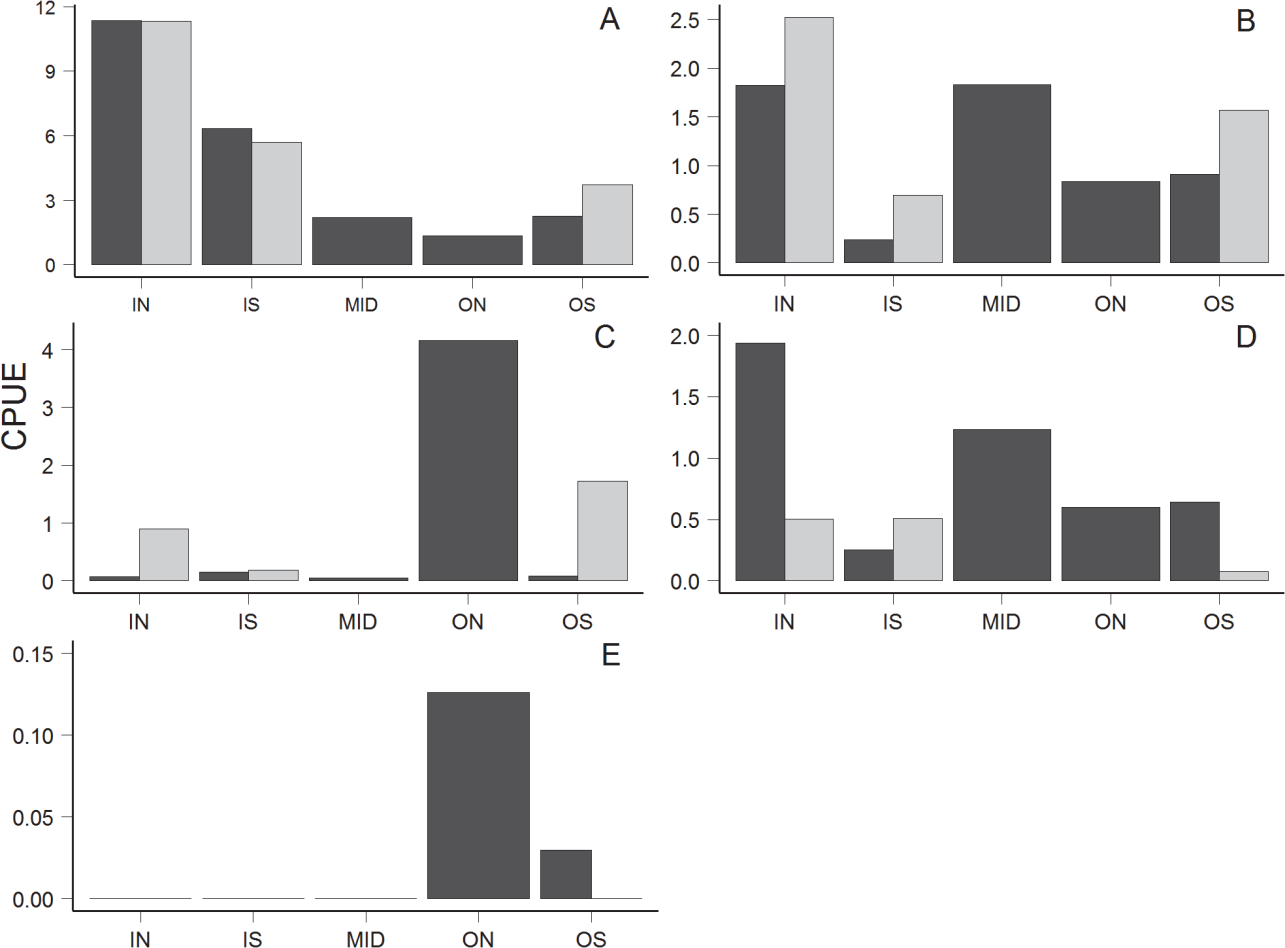
Average normalized trawl catch of all species of juvenile sockeye (a), coho (b), pink (c), Chinook (d) and chum (e) salmon, pooled across all locations and sampling dates and normalized for 20 min sets. Dark grey bars indicate 2007 and light grey bars indicate 2013. Note different scales for y‐axes for different species. Region boundaries and abbreviations are same as for Fig. 1.

The prevalence of each species of juvenile salmon varied by gear type, sub-region, and region (Figs. 4, 5). Sockeye salmon were not caught in beach seine sets but were abundant in nearshore trawls, in some cases within 20 m of shore. Pink salmon were most abundant in beach seine sets, especially at Kinahan Islands and at Ridley Island close to the proposed industrial developments (Fig. 4A). Most chum salmon were captured in the Tsum Tsadai Inlet area, outside of the proposed development footprints (Fig. 4B). The highest beach seine catches for coho and Chinook salmon were near proposed developments at Lelu and Ridley Islands (Fig. 4C and D). The highest abundances of juvenile sockeye salmon in trawl sets were captured in the IN region in both years (Fig. 5), the region containing proposed industrial developments. For regions that were sampled in both years, the abundances of juvenile sockeye and coho salmon captured by trawl were similar within regions across years (Fig. 5). The highest abundances of juvenile Chinook salmon were captured by trawl in the IN region in 2007, and evenly distributed between the IN and IS regions in 2013 (Fig. 5). In 2007, the highest abundances (mean normalized trawl catches for all weeks) of both pink and chum salmon were captured in the ON region (not sampled in 2013) at two of the northernmost sites close to Portland Inlet, which drains the Nass River and empties into Chatham Sound (Fig. 5C and E). In 2013, the highest abundances of pink salmon captured by trawl were found in the OS region (Fig. 5C).

**Fig 6:**
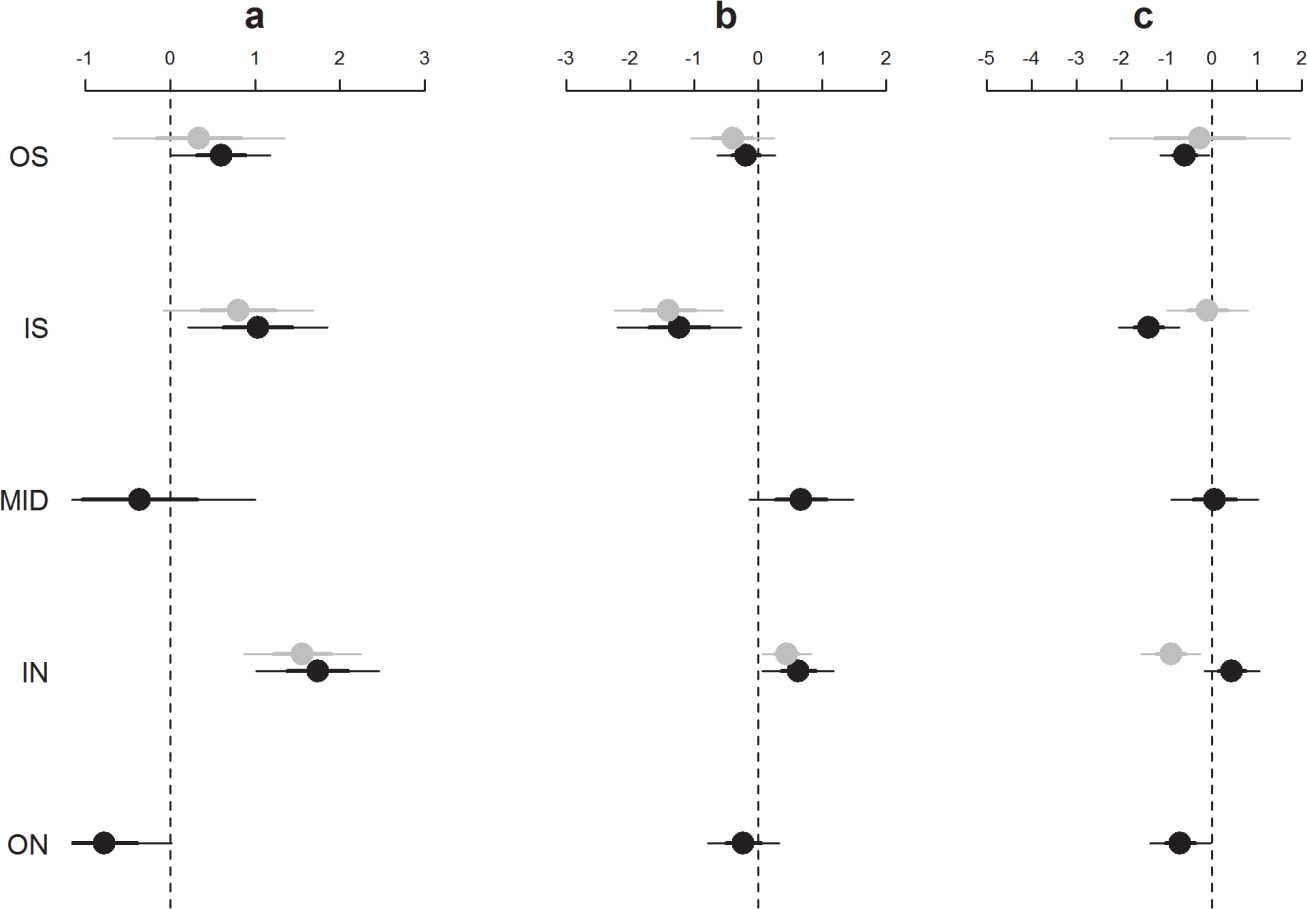
GAM coefficients for parametric region covariates for sockeye (a), coho (b) and Chinook (c) salmon. Coefficients are related to the (log) mean normalized catch per trawl set for each region in 2007 (black) and 2013 (grey ). Thus, a value of 0 indicates a mean normalized trawl catch of 1. Error bars indicate ± 2 standard errors.

Our observations of the relative abundances of the different salmon species among the different regions were supported by general additive modeling which accounted for seasonal variation. Specifically, the GAMs statistically indicated that juvenile sockeye salmon were most abundant in the IN region in both years, and juvenile coho salmon were most abundant in the IN region in 2013 (Fig. 6). The β coefficient for sockeye in the IN region was 1.74 ± 0.36 (p < 0.0001, this and the following represent the best estimate of the coefficient ± 1 SE and P-value of the coefficient) in 2007 and 1.56 ± 0.34 (p < 0.0001) in 2013 (Fig. 6). The predicted abundances for sockeye in the IN region were 2–8 x higher than in the other regions in both years. For example, the back transformed predicted abundances of sockeye for May 28 were 24 sockeye (per 20 minute set) in the IN, 11 in the IS, and 7 in the OS region in 2013, and 27 in the IN, 13 in the IS, 9 in the OS, 3 in the MID and 4 in the ON in 2007. The β coefficients for coho salmon in the IN region were 0.63 ± 0.28 (p = 0.0262) in 2007 and 0.45 ± 0.19 (p = 0.022) in 2013 (Fig. 6). The predicted abundances of coho were 2–7 x higher in the IN than in other regions in 2013, and 2–7 x higher in the IN and MID regions than in other regions in 2007. Chinook salmon appeared to be most abundant in the IN region in 2007 and in the IS region in 2013, however neither of these values were significant (p > 0.05). The delta-AIC score comparing each species-year model to an analogous version that excluded region was greater than 2, thus demonstrating support for including region for all year-species combinations except Chinook salmon in 2013. Because we sampled the top 4.5 meters of the water column over sites of varying depth, the juvenile salmon abundances are representative only of the surface layer of each region—this surface layer is where juvenile salmon are known to feed during the daylight hours in which we sampled [14,50].

### Genetic analysis

Genetic determinations were obtained from 476 sockeye salmon captured in 2007, 361 sockeye in 2013, and 19 Chinook salmon in 2013. Of these, 92% of the sockeye captured in 2007, 96% sockeye captured in 2013, and all of the Chinook originated in the Skeena watershed. If we consider only the highest precision genetic determinations, those for which probability of assignment exceeded 90%, five Chinook salmon populations were represented in ten individual fish, and at least seven of the remaining nine came from the more broadly defined Skeena watershed. Four of the five Chinook populations that exceeded the 90% probability threshold were captured in the IN region, including Chinook salmon from Nangeese River in the Kispiox sub-basin, Morice River, and Kitsumkalum River. A total of 220 individual sockeye salmon determinations from both years exceeded 90% probability, representing 25 individual populations including 15 from the Skeena, two from the Nass, and several from smaller coastal watersheds of the north and central coasts of British Columbia.

The highest genetic diversities for sockeye salmon were observed in the IN and OS regions. Twelve of the 13 different sockeye salmon populations that were captured in the IN region originated in the Skeena watershed, including populations from Alastair, Kitsumkalum, and Lakelse lakes in the lower Skeena, Morice Lake in the Bulkley system, Sustut Lake in the high interior, and several different populations of Babine Lake sockeye. Sockeye salmon from 14 different populations were captured in the OS region, of which eight were from the Skeena, and one was from the Kwinageese River in the Nass watershed. Several juvenile sockeye that were captured in the OS region originated from nearby coastal lakes including Lowe Lake in Grenville Channel and Freda and Kooryet Lakes on Banks Island. At least one sockeye salmon came from Namu Lake on BC’s central coast. Most of the specimens whose first probability of assignment did not exceed the 90% threshold were from the Babine drainage within the Skeena watershed (n = 548 of 616). The others, which were grouped by lake, sub-basin, watershed or statistical area came from the other Skeena sockeye lakes, coastal systems, and the Stikine drainage.

## Discussion

Our results indicate that the Skeena River estuary, especially the areas containing the proposed development footprints, supports diverse and abundant populations of juvenile salmon. During our two years of sampling, we found that the different species of juvenile salmon occupied the estuary from the middle of May until at least the beginning of July. Some of the highest abundances of some species were observed in areas proposed for development. Specifically, sockeye salmon were 2–8 times more abundant in the IN region compared with other regions in both years of sampling, coho salmon were 2–7 times more abundant in the IN and MID regions, and Chinook salmon were 2–6 times more abundant in the IN region in 2007. Juvenile Chinook and sockeye salmon were genetically identified as originating from populations throughout the Skeena watershed and beyond. These data provide evidence that the Skeena River estuary in general contains high abundances and population diversity of juvenile salmon. Within the greater Skeena estuary, the highest densities and highest population diversity of the most ecologically and economically important species of Skeena River salmon were found in the inside north region where development is proposed.

We captured thousands of juvenile pink salmon by beach seine within the proposed development footprints in the IN region. Pink salmon, which enter marine waters soon after emergence and return to spawn after a single year at sea, occupy estuarine habitats for several weeks as they gradually move further offshore [39]. For pink salmon, the earliest marine life-history stage is a critical period of high mortality [4], and the abundance of pink salmon smolts captured up to two months after emergence is used as an indicator to predict adult returns in the following year in Southeast Alaska [62]. Thus, adult salmon recruitment, and therefore the productivity of fisheries, may be determined by survival of juveniles during the early marine life-history stages such as those that we observed in our beach seine samples in the Skeena estuary. Juvenile chum salmon were also captured in high numbers in beach seine sets early in the sampling season. Several dozen larger juvenile chum salmon were captured in an experimental purse seine set in a nearby area in early August (Carr-Harris, unpublished), supporting the possibility that some populations may utilize these habitats for months [11,39], however further studies are required in order to determine the duration of estuarine residence and importance of these habitats for the species and populations of salmon that were captured in 2007 and 2013.

The region with proposed development contained the highest densities of juvenile sockeye and coho salmon in both years, and juvenile Chinook salmon in 2007. Abundances of sockeye and coho salmon were consistently higher in this region compared with other regions in the two years sampled, suggesting that the IN region contains consistently important rearing areas for out-migrating salmon smolts. These results are supported by historical studies of juvenile salmonids in the estuary of the Skeena River [39,40] that concluded that the areas currently proposed for development including the waters around Flora Bank and southeast Ridley Island are critical habitat for juvenile salmonids [48]. While these results are perhaps not surprising because it is well known that estuaries are important habitat for juvenile salmon [25,63,64], they differ from the recent reports by proponents’ consulting groups that have not reported significant numbers of juvenile salmon in this area [44–46]. The highest abundances of most species of juvenile salmon were observed within 10 km of the northern entrance of the Skeena River, either within the development footprints, or in habitats that juvenile salmon would have to transit through the proposed developments to access.

**Fig 7:**
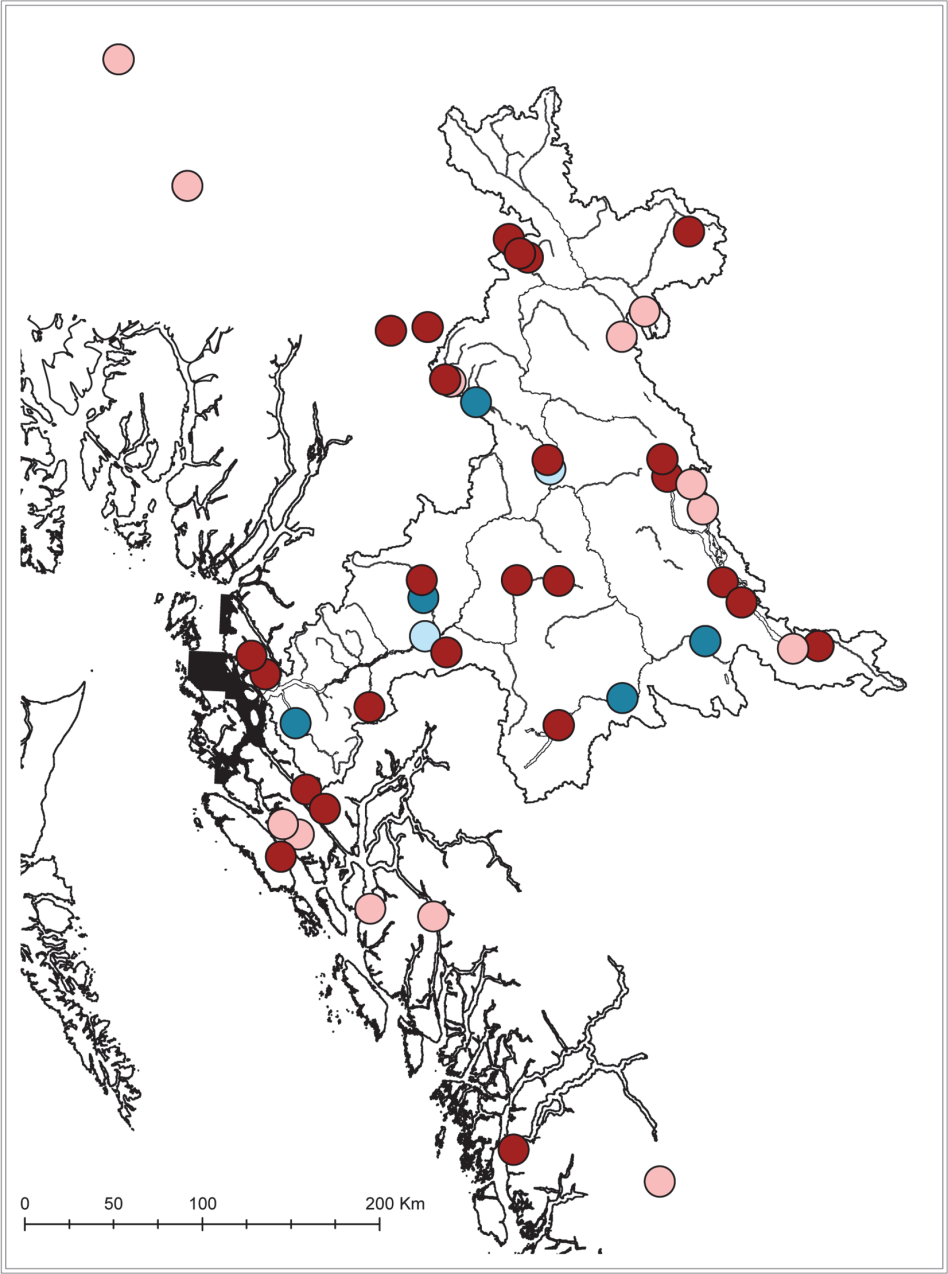
Map of the north coast of British Columbia and the Skeena watershed showing locations of origin for genetically identified sockeye and Chinook salmon smolts captured in the Skeena estuary in 2007 and 2013. Red and pink dots indicate the most likely location of origin for sockeye salmon, with locations that scored above (red) and below (pink) a 90% probability threshold for at least one specimen. Blue dots indicate the highest probability location of origin above (dark blue) and below (light blue) the 90% probability threshold for Chinook salmon. The sampling areas in the estuary of the Skeena River, where all fish were captured, are shown in black.

Our data indicate that the estuary of the Skeena River in general, and the area proposed for developments in particular, is an ecologically significant habitat that integrates diversity of all species of anadromous salmonids from the Skeena River and surrounding areas. We captured Chinook salmon from at least 5 different populations and sockeye salmon from 25 different populations from throughout the Skeena drainage and beyond, and sockeye salmon from most of the Skeena River populations currently represented in the DNA baseline. Specifically, 23 of the 29 sockeye salmon populations in the genetic baseline were represented in the probability distributions for genetic assignment for the combined 2007 and 2013 trawl samples, with over 90% probability of genetic assignment for 15 different Skeena sockeye salmon populations (Fig. 7). On a finer scale, the proposed development region contained particularly high salmon population diversity, with individual fish assigned to 13 sockeye salmon and 4 Chinook salmon populations. Some of the fish that we captured in this proposed development region are of conservation concern, such as sockeye salmon from Morice and Lakelse lakes and chum salmon from throughout the Skeena River watershed, for which low escapements in recent years compared with historical abundances have prompted calls for recovery planning [30]. Our data indicate that the Skeena estuary, especially the areas where development projects are proposed, represents important habitat for multiple salmon species and populations.

The salmon populations that we sampled near the proposed developments support important commercial, recreational, and First Nations fisheries. For example, we captured Chinook salmon from Morice River, which are targeted by the Wet’suwe’ten First Nation in Moricetown, approximately 450 km upriver from our capture sites, and from the Kitsumkalum River, approximately 120 km upriver, which are targeted by recreational fisheries in the Lower Skeena river and in coastal waters [27]. The majority of the sockeye salmon smolts from the proposed development zone were genetically identified as being from Babine Lake. Babine Lake sockeye are targeted by the Area 4 commercial gillnet fishery in Chatham Sound and the mouth of the Skeena River, a commercial terminal fishery in Babine Lake, and as well as First Nations food, social and ceremonial (FSC) fisheries that support thousands of individuals in 20 communities on the coast and along the Skeena River [27]. Fish that support fisheries are protected by the Fisheries Act, and First Nations fisheries are protected by the Canadian constitution [65]. Our data indicates that the estuary habitat is linked to fish that sustain a diversity of fisheries, warranting apparent protection under the terms of the revised Fisheries Act of 2013 [66] and the constitution [65].

Our research supports past research which suggests that the Skeena river estuary is utilized extensively by six species of diadromous Pacific salmon and steelhead during the critical marine entry stages, and may constitute a bottleneck. The term bottleneck has several meanings. First, migratory bottlenecks refer to areas along migratory routes where migration is constrained, leading to high abundances and diversity during migration [67]. For example, the oasis at Eilat (Elat), Israel is an important resting stop used by many species of Old World bird species during flights between Europe and sub-Saharan Africa [68]. Alternatively, a bottleneck is used to describe a specific phase of a life-cycle that limits overall productivity of population (e.g., [69,70]). Estuaries may act as both migratory bottlenecks as well as life-cycle bottlenecks for anadromous salmon. First, given the dendritic structure of river networks, the abundance and diversity of juvenile salmon from throughout river networks will necessarily be funneled through the base of the network. The Skeena estuary, which represents a small portion of the area of the vast Skeena watershed, funnels hundreds of millions of juvenile salmon through the transition from freshwater to marine habitats each year. Our study found that the estuary was utilized by salmon populations from throughout the Skeena estuary and beyond, together representing spawning areas ranging from local coastal streams to Sustut Lake in the high interior which is over 575 km inland from our sampling area. Second, the estuary and early marine period may also act as a life-history bottleneck. While mortality occurs throughout the life-cycle of anadromous salmon, past research indicates that the period upon marine entry is particularly important (eg. [4,6,7]). Thus, this area may represent a bottleneck that may control salmon productivity by geographical and biological means. The identification of either type of bottleneck can facilitate management or conservation activities.

Previous studies have found that anthropogenic alteration of estuary habitats can negatively impact juvenile salmon. Overwater structures such as piers or bridges impair juvenile salmon habitat usage and decrease their movement underneath these structures [71,72]. Such structures may also facilitate predation of juvenile salmonids. For instance, predation activity by cormorants and Caspian terns on juvenile Chinook in the Columbia River estuary was higher in areas near pile dykes, and the size of the bird colonies themselves increased following the formation of the artificially created Rice Island from disposed dredge material [73]. Juvenile salmon also have been found to exhibit preference for unaltered estuary habitat; for example, tagged juvenile Chinook salmon had a strong preference for and better survival in native eelgrass habitat compared to human-altered habitats (e.g., oyster aquaculture) [15]. Degradation of estuary habitat has also been found to be associated with population-level declines of salmon. A study of tagged juvenile Chinook and coho salmon over 37 years from 14 estuaries in the Puget Sound (Washington, USA) area found that Chinook salmon survival was 45% lower in estuaries contaminated by industrial pollutants [74]. A similar study found that survival of tagged Chinook salmon released in estuaries in the western states was significantly lower in more human-altered estuaries compared with pristine systems [19]. Other studies have found that juvenile salmonid rearing activities increased following the restoration of previously degraded estuaries [12,75]. For example, estuarine rearing opportunities for juvenile Chinook increased along with variation in life history strategies among subpopulations following removal of dikes in the Salmon River estuary [21]. There is evidence that estuary habitat degradation can negatively impact salmon populations.

Our study highlights the challenges of relying on proponent-funded research to assess potential environmental impacts of proposed developments. The environmental assessment studies conducted by project proponents provide an opportunity to collect information and identify important juvenile salmonid nursery habitats within the Skeena estuary; but the data collected on behalf of private industries are generally proprietary and inaccessible to independent review. For example, in 2010, a consulting organization on behalf of the proposed Canpotex potash terminal conducted fish sampling with similar gear, timing, and site selection as the current study. While the project’s eventual approval was based on an environmental impact statement that concluded that the juvenile salmon that had been observed at those sampling stations were not likely part of a major migration [44], these data were not disclosed to the public. In addition, recent changes to Canada’s environmental legislation may facilitate industrial development [76]. Economic co-dependency between industry and their private scientists will exert great pressure on the openness and integrity of environmental science [77]

**Fig 8:**
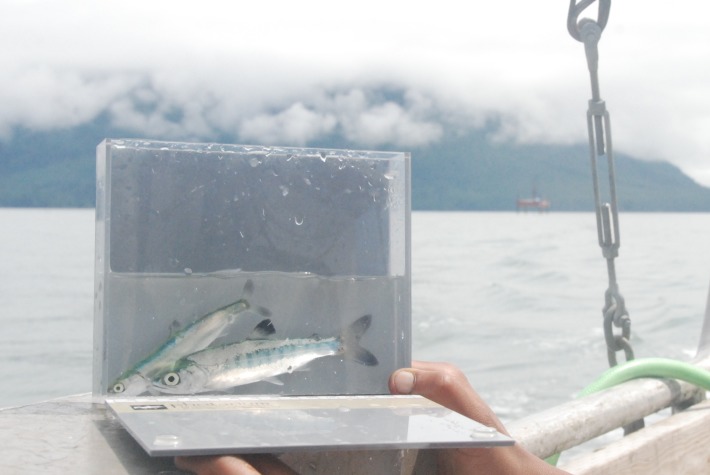
Picture of a pink salmon and a coho salmon smolt caught in the Skeena River estuary, in the area that is proposed to be dredged to accommodate tankers at a proposed terminal for natural gas. A drilling rig is in the background. Photo by J.W. Moore.

The Skeena River watershed is a region where annual salmon migrations sustain the ecosystem, culture, and economies of First Nations, commercial, and recreational fishing sectors [27]. Our data indicates that all surveyed parts of the estuary support salmon, especially the regions that are slated for development (Fig. 8). Proposed industrial projects would remove foreshore habitat, dredge benthic habitat, install causeways and berths, potentially mobilize contaminants in sediments, as well as increase tanker traffic. If industry projects are approved and proceed, and if these potential alterations to estuary habitats follow previously documented associations between estuary alteration and salmon declines (e.g., [19,74]), these alterations of this habitat could degrade nearby and distant fish populations and their fisheries.

## Supporting Information

S1 Table Trawl catch data from 2007. Locations, dates, trawl durations, and catches from trawl sampling from 2007.
(CSV)

S2 Table Trawl catch data from 2013. Locations, dates, trawl durations, and catches from trawl sampling from 2013.
(CSV)

S3 Table Beach seine data. Locations, dates and catches of beach seine sampling from 2013.
(CSV)

S4 Table Chinook salmon genetic population identification data. Data on juvenile Chinook salmon, their capture region and the probability of assignment for the top five possible populations. All of these fish were collected during 2013.
(CSV)

S5 Table Sockeye salmon genetic population identification data. Data on juvenile sockeye salmon, their year and region of capture and the probability of assignment for the top five possible populations.
(CSV)
